# Porous Scaffold-Hydrogel Composites Spatially Regulate 3D Cellular Mechanosensing

**DOI:** 10.3389/fmedt.2022.884314

**Published:** 2022-05-02

**Authors:** Matthew DiCerbo, Mohammed Mehdi Benmassaoud, Sebastián L. Vega

**Affiliations:** Department of Biomedical Engineering, Rowan University, Glassboro, NJ, United States

**Keywords:** 3D hydrogels, cellular mechanosensing, cellular remodeling, hydrogel, tissue interface, 3D biomaterials, stem cells, osteochondral interface

## Abstract

Cells encapsulated in 3D hydrogels exhibit differences in cellular mechanosensing based on their ability to remodel their surrounding hydrogel environment. Although cells in tissue interfaces feature a range of mechanosensitive states, it is challenging to recreate this in 3D biomaterials. Human mesenchymal stem cells (MSCs) encapsulated in methacrylated gelatin (GelMe) hydrogels remodel their local hydrogel environment in a time-dependent manner, with a significant increase in cell volume and nuclear Yes-associated protein (YAP) localization between 3 and 5 days in culture. A finite element analysis model of compression showed spatial differences in hydrogel stress of compressed GelMe hydrogels, and MSC-laden GelMe hydrogels were compressed (0–50%) for 3 days to evaluate the role of spatial differences in hydrogel stress on 3D cellular mechanosensing. MSCs in the edge (high stress) were significantly larger, less round, and had increased nuclear YAP in comparison to MSCs in the center (low stress) of 25% compressed GelMe hydrogels. At 50% compression, GelMe hydrogels were under high stress throughout, and this resulted in a consistent increase in MSC volume and nuclear YAP across the entire hydrogel. To recreate heterogeneous mechanical signals present in tissue interfaces, porous polycaprolactone (PCL) scaffolds were perfused with an MSC-laden GelMe hydrogel solution. MSCs in different pore diameter (~280–430 μm) constructs showed an increased range in morphology and nuclear YAP with increasing pore size. Hydrogel stress influences MSC mechanosensing, and porous scaffold-hydrogel composites that expose MSCs to diverse mechanical signals are a unique biomaterial for studying and designing tissue interfaces.

## Introduction

Mesenchymals stem cells (MSCs) are highly sensitive to biochemical and biophysical signals (1, 2), and can differentiate into several musculoskeletal lineages including osteoblasts and chondrocytes (3). Hydrogels are soft biomaterials that have gained increased interest as matrices for tissue engineering due to their biocompatibility and ease of cellular encapsulation for 3D culture (4, 5). Hydrogels can also be designed with tunable biophysical properties including degradation (6, 7), which can be used to regulate 3D cellular spreading and mechanosensing of encapsulated cells (7). MSCs encapsulated in non-degradable hydrogels that are physically (e.g., alginate) (8) or covalently crosslinked (e.g., hyaluronic acid) (9) adopt a spherical morphology due to their entrapment in nano-porous hydrogel networks (10). For cells to spread inside 3D hydrogels, they need to remodel their surrounding hydrogel material, either by physically breaking reversible bonds (e.g., shear-thinning hydrogels) (11) or by enzymatically cleaving moieties in hydrogel crosslinkers or macromers. Cellular spreading is closely related to cellular mechanosensing, which is the ability for cells to sense biophysical properties of their surrounding environment by converting external inputs into actionable outcomes. Cellular mechanosensing is regulated by several mechano-transducer proteins, and of these YAP (Yes-associated protein) is a mechanosensitive protein that relays external signals to the nucleus (12), and its signaling is influenced by numerous environmental cues including stiffness, dimensionality, and cellular spreading (7, 12, 13).

Although degradable hydrogels are amenable to cellular remodeling and regulate 3D cellular mechanosensing, the use of these materials for tissue repair and regeneration is limited by the challenge of designing hydrogels with mechanical properties comparable to many native tissues. To enhance mechanics of single-network (e.g., methacrylated gelatin) hydrogels without compromising the viability of encapsulated cells, alternate crosslinking strategies involving two or more independent networks to form hydrogels have been explored (14). Hydrogels fortified with polycaprolactone (PCL) have also shown promise for developing biomaterials with mechanics comparable to cartilage and bone. For example, the mechanical properties of soft GelMe hydrogels can match and even exceed the compressive modulus of native articular cartilage by reinforcing with highly porous PCL microfiber networks (15). The combination of 3D printing of PCL structures, cells, and cell-laden GelMe hydrogels has also been explored to recreate the biophysical environment of osteochondral tissue interfaces (16).

PCL-hydrogel composites not only increase bulk mechanics but also introduce local changes to resident cells within the hydrogel network. Cells have the remarkable ability to sense material stiffness across a much softer medium (17), and cells are sensitive to topographical features of rigid surfaces (18). Diverse PCL structures can be achieved by various techniques including 3D printing, electrospinning, and particle-leaching, and this study reports the development of porous PCL-hydrogel composites to steer cells toward different mechanosensitive states present in tissue interfaces. Methacrylated gelatin (GelMe) is an MMP-sensitive collagen-derived macromer with adhesive domains (19, 20), and MSCs encapsulated in GelMe hydrogels significantly remodel their local hydrogel environment between 3 and 5 days in culture. Using a custom compression device, MSC-laden hydrogels were compressed for 3 days, and MSCs in edge regions (high compressive stress) displayed increased mechanosensing when compared to MSCs in center regions (low compressive stress). MSC populations in porous scaffold-hydrogel composites were evenly distributed and the diversity in morphology and YAP signaling varied based on the local PCL microarchitecture.

## Materials and Methods

### Materials

Gelatin from porcine skin (Type A, ~300 g Bloom), methacrylate anhydride, polycaprolactone (PCL), Irgacure 2959 (I2959), triethanolamine, and SpectraPor 12–14 kDa molecular weight cutoff (MWCO) dialysis tubes were purchased from Millipore Sigma (St. Louis, MO, USA). Phosphate buffered saline (PBS), sodium chloride (NaCl), and dichloromethane (DCM) were purchased from VWR International (Wayne, PA, USA). Polydimethylsiloxane (Sylgard 184, PDMS) and 8 mm biopsy punches (Accu-Punch) were purchased from Electron Microscopy Sciences (Hatfield, PA, USA). Minimum Essential Medium (αMEM), fetal bovine serum (FBS), and penicillin/streptomycin (P/S) were purchased from Gibco (Waltham, MA). Stains for actin (phalloidin, Alexa Fluor 488) and double stranded DNA (Hoescht 33342) were purchased from Invitrogen (Carlsbad, CA, USA). Primary YAP antibody (SC-376830) was purchased from Santa Cruz Biotechnology (Santa Cruz, CA, USA) and secondary antibody (Alexa Fluor 568) was purchased from Invitrogen (Carlsbad, CA, USA).

### GelMe Macromer Synthesis

GelMe was synthesized using a modified synthesis scheme previously described (19, 20). Briefly, 10 wt% gelatin was dissolved in a PBS stir bath (50°C, 1,200 RPM) for 20 min. 5 wt% of methacrylate anhydride was added to the gelatin solution at a rate of 0.5 ml/min while stirring at 50°C and was reacted for 1 h. The reaction was quenched by adding excess (4x) prewarmed PBS and GelMe was dialyzed (SpectraPor, 12–14 kDa MWCO) for 5 days at 40°C, frozen, and lyophilized. The degree of methacrylation was determined with ^1^H NMR spectroscopy and was found to be 58.2% (Supplementary Figure 1).

### Hydrogel Synthesis and Characterization

GelMe solutions (5 wt% GelMe and 0.05 wt% photoinitiator I2959 in PBS) were pipetted into PDMS cylindrical molds (8 mm diameter, 2 mm height) and photopolymerized with UV light (10 mW/cm^2^, 10 min). For compression testing, GelMe hydrogels were incubated in PBS overnight at 37°C and compressed with a Shimadzu EZ-SX Mechanical Tester up to 30% strain at a strain rate of 10%/min. The elastic modulus was calculated using the slope of the formed stress-strain curve between 10 and 20% strain.

### Cell Culture

Human bone marrow-derived MSCs (Lonza, PT-2501, Lot 684888, 33-year-old male) were cultured in 100 mm cell culture dishes in Growth Medium (αMEM supplemented with 10% FBS and 1% P/S) in a humidity-controlled environment under 5% CO_2_ and 37°C. MSCs were passaged upon reaching 80% confluence and MSCs were used at passage 2 or passage 3 for all experiments.

### MSC-Laden GelMe Hydrogel Culture

MSC-laden GelMe solutions (1E6 MSCs/ml, 5 wt% GelMe, and 0.05 wt% I2959 in Growth Medium) were pipetted into PDMS cylindrical molds (8 mm diameter, 2 mm height) and photopolymerized with UV light (10 mW/cm^2^, 10 min). Previous studies using comparable photopolymerization settings to crosslink hydrogels showed no impact on viability or cell function of encapsulated cells (6, 21). To evaluate cellular remodeling kinetics, free-swelling hydrogels were cultured in Growth Medium for 1, 3, 5, or 7 days and stained for actin (phalloidin 40 min, 1:100), YAP (primary 1:200, overnight; secondary 1:200, 2 h), and double stranded DNA (Hoescht, 5 min, 1:2,500). Samples were then imaged with a Nikon A1 confocal microscope and images were analyzed with ImageJ (Version 1.53j) software (NIH, Bethesda, MD, USA).

To evaluate the effects of compression on spatial differences of MSC morphology and YAP signaling, a custom compression device was developed (Supplementary Figure 2). The device supports a standard 24-well plate within it, and it can maintain up to 24 hydrogel samples under varying degrees of compression using nylon bolts with a cylindrical head cap. To compress, MSC-laden GelMe hydrogels were affixed to thiolated coverslips which were then placed in wells of a 24-well plate. A nylon bolt then was then lowered from above until it contacted the top of the hydrogel and was rotated (1/6 rotation = 325 μm) until 25 or 50% compression was achieved. Hydrogels were held under compression for 3 days prior to fixation and staining. To determine the Edge and Center regions for imaging and analysis, stained hydrogels were placed on a thin glass coversip and the edge of the hydrogel along the x-axis (left to right) was identified. From there, the hydrogel was separated into Edge regions (distance 0–2 mm and 6–8 mm from the edge in the x-direction) and a Center region (distance 2–6 mm from the edge in the x-direction) for imaging and analysis.

### Porous Scaffold Fabrication and Characterization

Porous PCL scaffolds were formed using a salt-leaching technique outlined in Supplementary Figure 3. Briefly, 30 wt% PCL was dissolved in 70 wt% DCM. Salt crystals (NaCl) with diameters ranging between 212 and 500 μm were acquired using salt sieves. PCL solutions were mixed with either 8:1, 12:1, or 15:1 NaCl-to-PCL weight ratio representing Low, Med, and High porosity, respectively. The NaCl/PCL mixture was poured into a mold, flattened, and DCM was allowed to evaporate for 24 h in a fume hood. Post-DCM evaporation, a biopsy punch (8 mm diameter) was used to create cylindrical scaffolds, which were then placed in conical tubes containing purified water in a revolver lab rotator for 5 days (water changed 2x/day). This last step forms PCL scaffold porosity due to salt crystals leaching out of the bulk PCL scaffold.

### Hydrogel Perfusion Into Porous Scaffolds

GelMe solutions (1E6 MSCs/ml, 5 wt% GelMe, and 0.05 wt% I2959 in Growth Medium) were perfused into porous PCL scaffolds (Supplementary Figure 4). Briefly, GelMe solution (500 μl) was added to a 3 ml Luer lock syringe and loaded onto a syringe pump. The needle was inserted into a segment of sterile Tygon tubing (8 mm inner diameter), containing the porous scaffold. All connections were secured with parafilm. GelMe solution was then perfused into the porous scaffold at a rate of 1 ml/min, the porous scaffold-hydrogel composite was carefully removed from the Tygon tubing, and the GelMe within the construct was photopolymerized with UV light (10 mW/cm^2^, 5 min per side). Samples were then placed in wells of a 24-well plate, washed in Growth Medium 3 times, and incubated in Growth Medium for 3 days. Lastly, samples were stained for actin, YAP, and double stranded DNA, imaged with a Nikon A1 confocal microscope, and images were analyzed with ImageJ software.

### Image Analysis

Image analysis was performed for single cells using ImageJ software. For each cell, three metrics were calculated: Cellular Volume, Sphericity, and Nuclear YAP. Cellular Volume and Sphericity (line and sphere have values of 0 and 1, respectively) for each cell were determined by converting 3D image stacks of the actin channel into binary objects using Otsu's intensity-based thresholding method, followed by ImageJ's 3D Objects Counter function. Nuclear YAP was determined by converting 3D image stacks of the actin and nuclei channels into binary objects, which were then superimposed with the YAP channel to get 3D image stacks of cytosolic and nuclear YAP. Using ImageJ's 3D Objects Counter function, the sum of pixel intensity values for the nuclear and cytosolic YAP was determined and normalized to nuclear and cytosolic volumes. Then, nuclear YAP was calculated by taking the ratio of normalized nuclear to cytosolic YAP.

### Statistical Analysis

**Figure 1:**
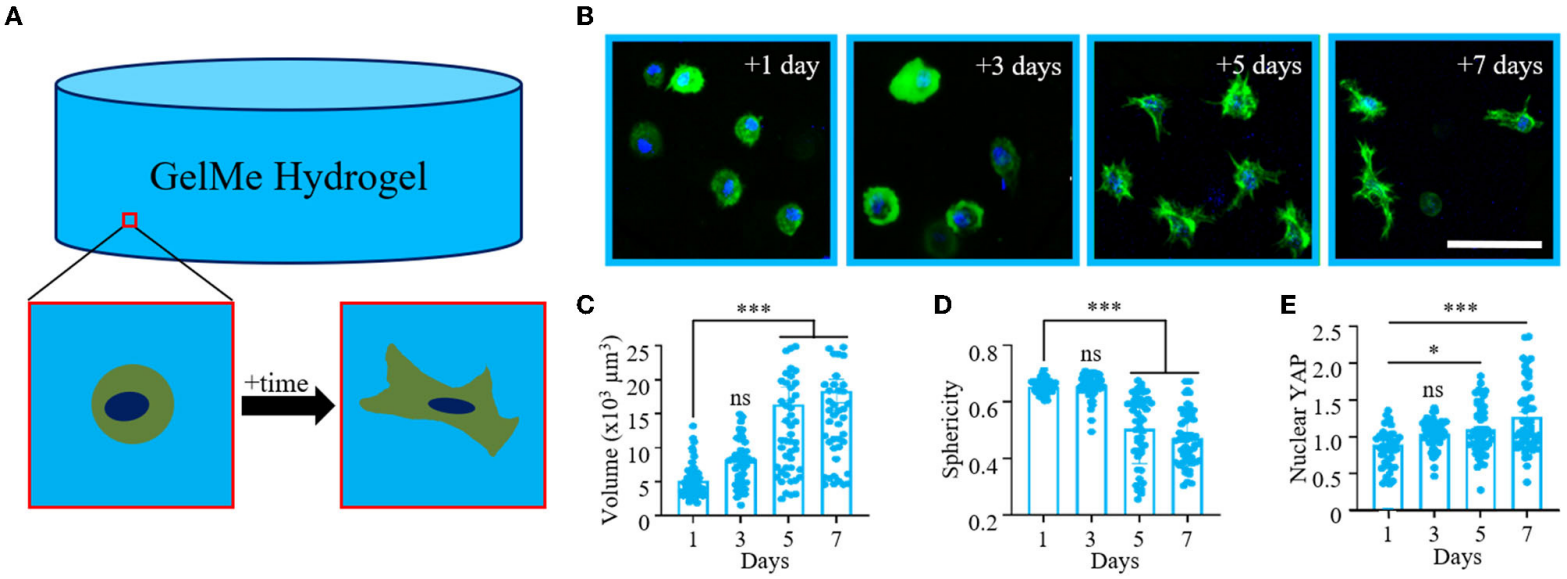
Temporal changes in MSC morphology and YAP signaling in 3D GelMe hydrogels. **(A)** Schematic of a single MSC encapsulated in a GelMe hydrogel remodeling its local hydrogel environment over time. **(B)** Representative confocal images of MSCs (green, actin; blue, nuclei) encapsulated in GelMe hydrogels for 1, 3, 5, and 7 days. Quantification of cellular **(C)** Volume, **(D)** Sphericity, and **(E)** Nuclear YAP of MSCs encapsulated in GelMe hydrogels after 1, 3, 5, and 7 days in culture. Bar graphs represent the mean and points individual cells; *n* > 50 cells per group, ns, not significant, ^*^p < 0.05, ^***^p < 0.001. Scale bar: 100 μm.

**Figure 2:**
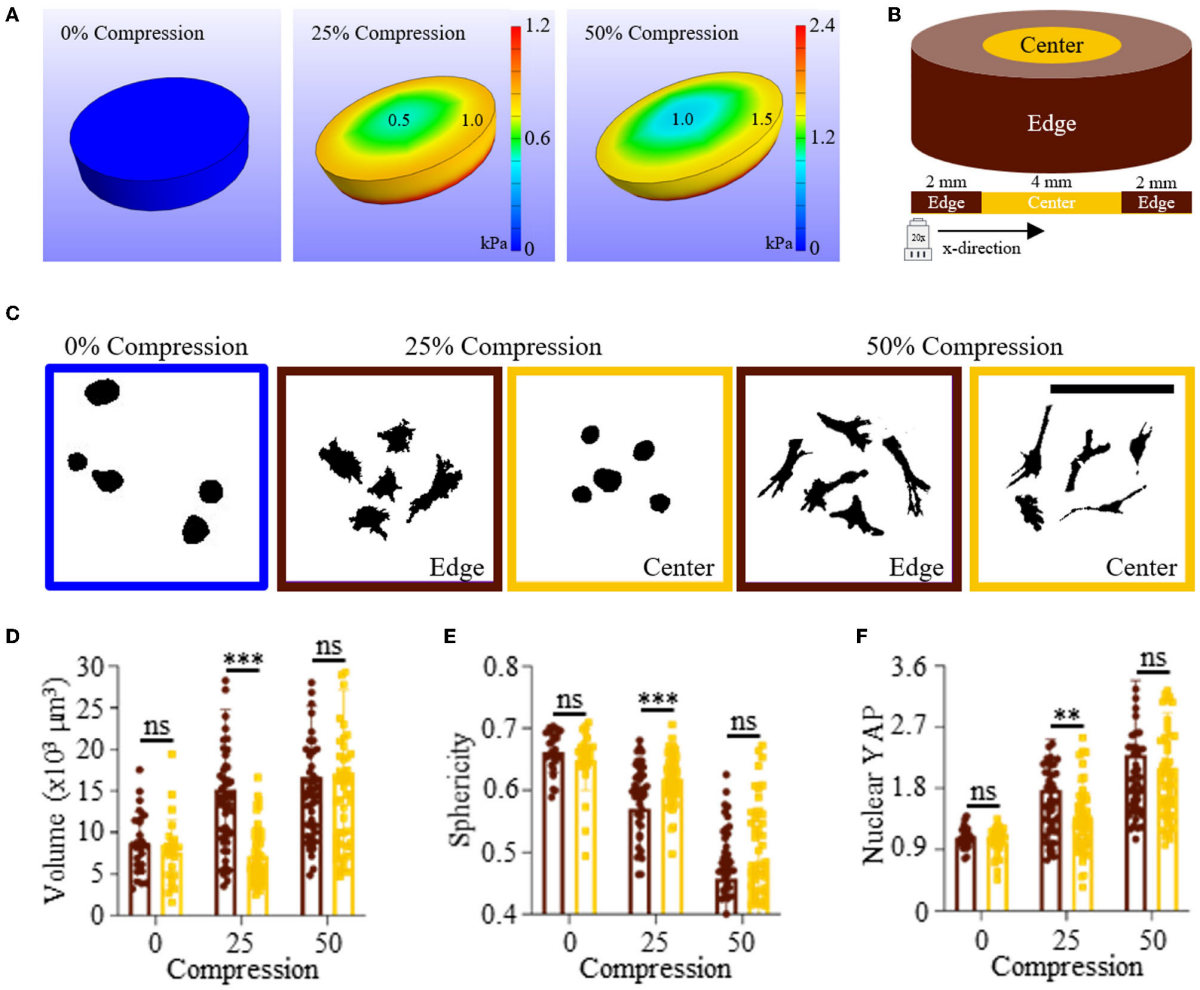
Differences in hydrogel stress during compression influence MSC spreading and nuclear YAP localization in GelMe hydrogels. **(A)** Finite element analysis models of uncompressed (left), 25% (middle), and 50% (right) compressed hydrogels. **(B)** Schematic of hydrogel showing edge (brown) and center (gold) regions for the whole hydrogel and along the x-direction. **(C)** Representative single cell silhouettes of single MSCs encapsulated in uncompressed, 25% compressed, and 50% compressed GelMe hydrogels. Quantification of cellular **(D)** Volume, **(E)** Sphericity, and **(F)** Nuclear YAP of MSCs encapsulated in edge (brown) or center (gold) GelMe hydrogels undergoing 0, 25, or 50% compression. Bar graphs represent the mean and points individual cells; *n* > 50 cells per group, ns, not significant, **p < 0.01, ***p < 0.001. Scale bar: 100 μm.

**Figure 3:**
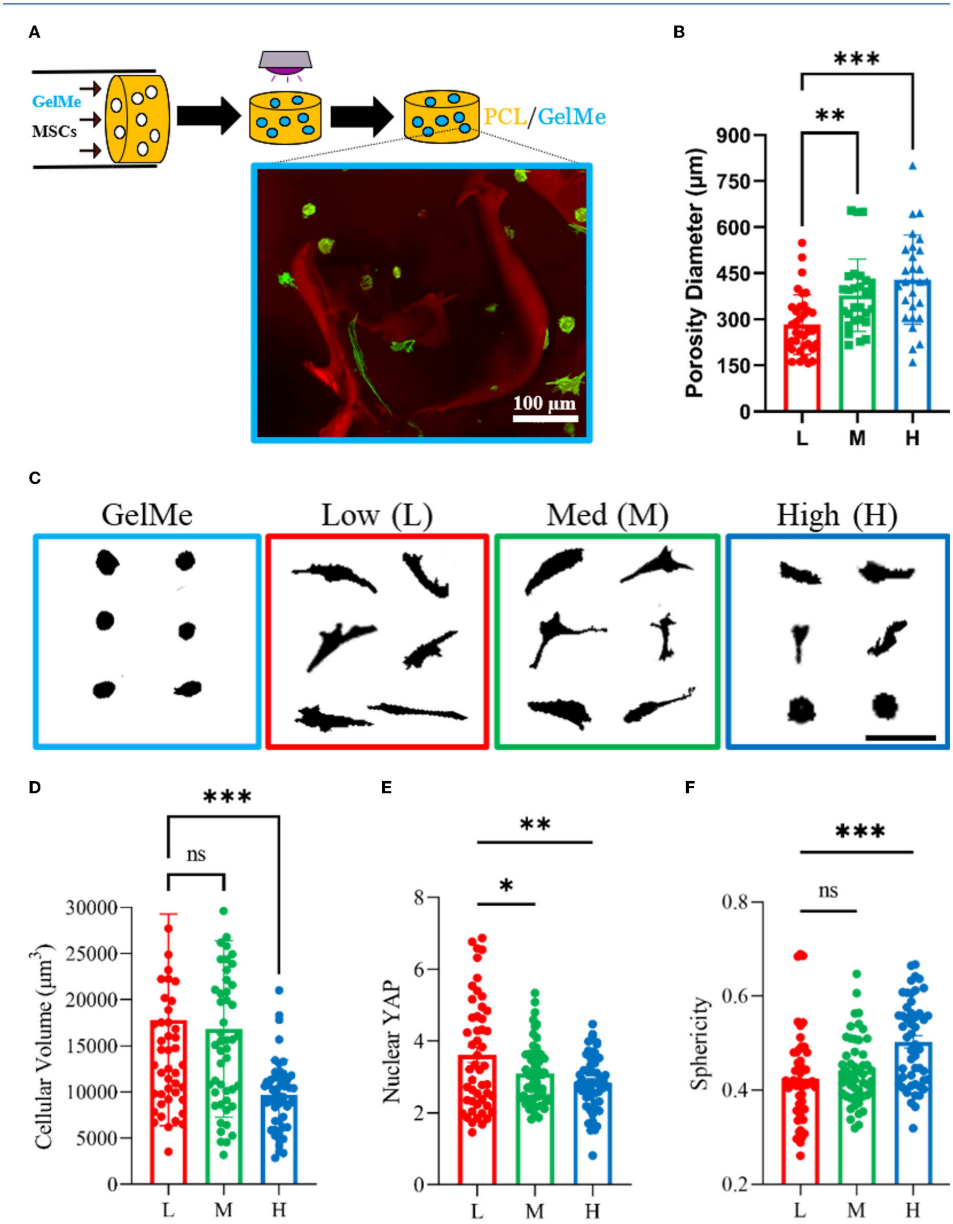
Pore diameter affects MSC mechanosensing in porous scaffold-hydrogel composites. **(A)** Schematic of MSC-laden GelMe perfusion process and representative confocal image of encapsulated MSCs (green) interacting with proximal pores (red). **(B)** Quantification of pore diameters for Low (L), Med (M), and High (H) porosity scaffolds. **(C)** Representative single cell silhouettes of MSCs encapsulated in GelMe and in GelMe perfused into L, M, and H porosity scaffolds. Quantification of cellular **(D)** Volume, **(E)** Nuclear YAP, and **(F)** Sphericity of MSCs encapsulated in GelMe perfused into L, M, and H porosity scaffolds. Bar graphs represent the mean and points individual cells; *n* > 50 cells per group, ns, not significant, ^*^p < 0.05, ^**^p < 0.01, ^***^p < 0.001. Scale bars: 100 μm.

All MSC experiments were carried out in at least three independent hydrogel (Figures 1C–E, 2D–F) and porous scaffold-hydrogel composite (Figures 3D–F) replicates. All single cell analyses were performed with at least 50 cells per group. For compression testing of hydrogel and porous scaffold-hydrogel composites (Supplementary Figure 5), at least 6 constructs were measured per group. Pore diameter measurements (Figure 3B) were from at least 6 porous scaffolds, and at least 50 individual pores were measured per group. Bar graphs represent mean ± standard error of the mean and values reported in the text are average ± standard deviation (SD). GraphPad Prism 9 was used for all statistical analysis (GraphPad Software Inc. San Diego, CA, USA). Analysis of variance (ANOVA) followed by Tukey's *post-hoc* test was performed on all data sets. Significance is indicated by ns (no significance, *p* > 0.05), ^*^p < 0.05, ^**^p < 0.01, and ^***^p < 0.001.

## Results and Discussion

### 3D Cellular Remodeling of MSCs in GelMe Hydrogels Is Time-Dependent

MSCs encapsulated in GelMe hydrogels are able to spread via MMP degradation (19, 20), and this is a time-dependent process (Figure 1A). For MMP-mediated hydrogel remodeling, the rate and extent of 3D cellular spreading is dependent on the concentration of degradable material. For instance, MSCs encapsulated in hyaluronic acid hydrogels crosslinked with a low concentration of MMP-peptides spread after 1 week in culture, whereas MSC spreading is restricted at higher MMP-peptide concentrations (7). To determine how long it takes encapsulated MSCs to degrade and remodel GelMe hydrogels, MSCs (1E6 cells/ml) were encapsulated in GelMe hydrogels (5 wt%) for 1, 3, 5, or 7 days. At each time-point, resident MSCs were evaluated for changes in morphology and YAP signaling. At 1- and 3-day time-points, MSCs retained a spherical morphology. However, between 3 and 5 days, MSCs began to extend protrusions indicative that local cellular remodeling was taking place (Figure 1B). After 1 day in culture, MSCs encapsulated in GelMe hydrogels had an average volume of 5,100 ± 2,500 μm^3^, and at 3 days there was a non-significant increase in MSC volume to 8,200 ± 3,800 μm^3^. Between 3 and 5 days, there was a significant increase in cell volume to 14,200 ± 8,900 μm^3^, and between 5 and 7 days the volume increased even further, to 18,300 ± 12,200 μm^3^ (Figure 1C). It is interesting to note that not only did the average cell volumes increase over time, but so did the standard deviation. This suggests that initially, MSC remodeling is highly consistent on a per-cell basis, and once cells surpass a threshold of remodeling, the ability (or inclination) for cells to continue spreading varies significantly on a single-cell level with increasing cell culture time.

Between 1 and 3 days in culture, there was a non-significant change in sphericity (0.65 ± 0.02 vs. 0.66 ± 0.04). However, between 3 and 5 days in culture, there was a significant decrease in sphericity, to 0.50 ± 0.12. As cells continued to spread, there was slight drop in sphericity, with 7-day average measured as 0.47 ± 0.10 (Figure 1D). Sphericity did not significantly change, with average values of 0.65 and 0.66 after 1 and 3 days in culture. As expected, nuclear YAP also increased with time, with a significant change observed between 1 and 5 days and 1 and 7 days in culture (Figure 1E). Taken together, these findings show that in unconfined GelMe hydrogels it takes MSCs at least 3 days to significantly remodel their surrounding gelatin material, leading to significant changes in cell morphology and YAP signaling. Further, the range of MSC metrics measured here (Volume, Sphericity, Nuclear YAP) increased with increasing culture time.

### MSC Morphology and YAP Signaling Varies Spatially in Compressed GelMe Hydrogels

Cells in compressed hydrogels can sense local changes in hydrogel stress induced by an applied force (22). For example, chondrocytes encapsulated in hydrogels under dynamic compression produced more cartilage extracellular matrix in comparison to chondrocytes in unconfined hydrogels (23). To determine the extent in which uniaxial compression causes regional changes in intra-hydrogel stress, a finite element analysis model of an elastic hydrogel under compression was developed using FEBio software (methods and model parameters in Supplementary Material). At 0% compression, the cylindrical hydrogel render did not show any stress, as expected. At 25% compression, a concentric gradient of stress was observed, with higher levels of stress in the edge region (~1 kPa) vs. the center region (~0.5 kPa). At 50% compression, the same hydrogel stress trend was observed, however, the values were significantly higher. The center (low stress) of the 50% compressed hydrogel had stress of 1 kPa, which was the same value of the edge (high stress) of the 25% compressed hydrogel model (Figure 2A). Based on these findings, it was hypothesized that encapsulated MSCs would be larger, less spherical, and feature higher YAP signaling in edge regions of compressed hydrogels, due to increased hydrogel stress from an applied compressive force.

To test this hypothesis, a custom uniaxial compression device was developed (Supplementary Figure 2). MSC-laden GelMe hydrogels (1E6 cells/ml) were formed in cylindrical molds affixed onto thiolated glass coverslips, and cylindrical molds were carefully removed prior to transferring samples to wells of a 24-well plate. Culture wells were filled with Growth Medium and placed in the compression device. A nylon bolt was then lowered from above until it compressed the hydrogel 0, 25, or 50% of its free-swelling height for 3 days. After 3 days, samples were fixed, stained, and MSC morphology and YAP signaling was evaluated in edge or center regions of the hydrogel samples (Figure 2B). Representative silhouettes of single MSCs show that at 0% compression, MSCs retain a spherical morphology after 3 days in culture. Although a spherical morphology is also observed for the center (low stress) region of 25% compressed hydrogels, MSCs spread in edge (high stress) regions of these hydrogels. MSCs in 50% compressed hydrogels had the same highly elongated and spread morphology in center and edge regions (Figure 2C). This could be due to the larger applied compressive force, since the finite element analysis model of compression predicted that at 50% compression the lowest region of stress (center) would equal the highest region of stress (edge) for 25% compressed hydrogels.

An edge vs. center region comparison of MSC volume shows that MSCs in the edge region of 25% compressed hydrogels were significantly larger than those in the lower stress center region. MSC volume in unconfined (0%) and highly (50%) compressed hydrogels was the same in edge and center regions (Figure 2D). Sphericity was the largest for MSCs in uncompressed GelMe hydrogels, and lowest in 50% compressed hydrogels. Concomitant with volume, MSCs were significantly less round in the edge region when compared to the center region of 25% compressed hydrogels (Figure 2E). Nuclear YAP of MSCs in uncompressed GelMe hydrogels was also the lowest after 3 days in culture (1.04 ± 0.19), whereas regional differences were only observed in 25% compressed hydrogels. MSCs in 50% compressed hydrogels had the highest levels of nuclear YAP (2.30 ± 1.1 in edge, 2.10 ± 0.8 in center) (Figure 2F). Nuclear YAP in highly compressed hydrogels is over two-fold higher than in uncompressed hydrogels, which suggests that confining hydrogels into tightly packed regions will induce changes in mechanosensitive states comparable to those present in tissue interfaces.

### Diversity in MSC Morphology and Mechanosensing Observed in Porous Scaffold-Hydrogel Composites

Based on the findings presented and previous studies on cell-hydrogel mechanosensing (17, 22, 24), it is hypothesized that MSC-laden GelMe hydrogels inside rigid yet porous PCL scaffolds will feature a range of hydrogel stresses which will translate to diverse mechanosensitive states. To test this hypothesis, MSCs in GelMe solution were perfused into porous PCL scaffolds and cultured for 3 days (Figure 3A). A representative confocal image of GelMe-encapsulated MSCs (green) in a red-labeled porous PCL scaffold display spherical and spread MSCs based on their proximity to rigid PCL pores (Figure 3A, inset). Using a salt-leaching technique, PCL scaffolds with Low (L, 280 ± 100 μm), Med (M, 325 ± 210 μm), and High (H, 430 ± 145 μm) pore diameters were developed (Figure 3B). The composites had a significantly higher compressive modulus than GelMe alone. The compressive modulus of GelMe hydrogels is 7.15 ± 0.8 kPa, whereas composites made with Low pore diameter are 570 ± 60 kPa. Increasing porosity resulted in a decrease in compressive modulus, with values for Med and High pore diameter composites as 300 ± 40 kPa and 100 ± 20 kPa, respectively (Supplementary Figure 5). MSCs were highly viable in the composites, with at least 80% viability across all constructs after at least 7 days in culture (Supplementary Figure 6).

MSC mechanosensing and population heterogeneity can be controlled depending on pore diameter. On Low pore diameter composites, MSCs are in closest proximity to PCL, and a large fraction of cells elongate due to being within small pores that are always close to the rigid PCL (Figure 3C, red box). On Med pore diameter composites, the pores are slightly larger and although most cells are spread, cell morphology is more diverse (Figure 3C, green box). In contrast, on High pore diameter composites, MSCs are either in proximity (near PCL/hydrogel interface) or further away (e.g., suspended in GelMe at the center of the larger pores), resulting in round and spread morphologies (Figure 3C, blue box). Quantification of cell volume shows that cell volume decreases with increasing pore diameter (Figure 3D). Similarly, Nuclear YAP decreases with increasing pore size (Figure 3E), and Sphericity increases with increasing PCL porosity (Figure 3F).

## Conclusions

Cells can “feel” biophysical properties of their surroundings, and this unique ability can be leveraged to design biomaterials that control cell behavior. This study shows that degradable elastic hydrogels under compression exhibit regional differences in material stress, leading to local differences in stem cell morphology and nuclear YAP localization. By perfusing cells and hydrogel solution into porous scaffolds, soft hydrogel-rigid scaffold interactions lead to a range in 3D mechanosensitive states of resident stem cells, which can be controlled by changing pore size. In this Brief Research Report, one MSC donor was used, and conducting these cellular mechanosensing studies with additional donors would strengthen the reported findings. The biological readouts of this study were early changes in morphology and nuclear YAP localization, and the effects of cellular mechanosensing on long-term stem cell differentiation within these composites is an area yet to be explored. Furthermore, by changing the hydrogel chemistry used, additional signals within these composites can be independently tuned. Norbornene-modified hydrogels can be photopatterned with thiolated peptides (21, 25), thereby introducing biochemical signaling as an additional input for controlling cellular mechanosensing and stem cell differentiation.

## Data Availability Statement

The raw data supporting the conclusions of this article will be made available by the authors, without undue reservation.

## Author Contributions

MD, MB, and SV designed the research, interpreted data, and wrote the manuscript. MD and MB designed experiments and performed analysis. MD designed the hydrogel compression device used in this study. All authors contributed to the article and approved the submitted version.

## Funding

This work was financially supported by a grant from the National Science Foundation (DMR-2037055).

## Conflict of Interest

The authors declare that the research was conducted in the absence of any commercial or financial relationships that could be construed as a potential conflict of interest.

## Publisher's Note

All claims expressed in this article are solely those of the authors and do not necessarily represent those of their affiliated organizations, or those of the publisher, the editors and the reviewers. Any product that may be evaluated in this article, or claim that may be made by its manufacturer, is not guaranteed or endorsed by the publisher.
