# Supplementary material for: Porous Scaffold-Hydrogel Composites Spatially Regulate 3D Cellular Mechanosensing
Source: Front Med Technol. 2022 May 2;4:884314. doi: 10.3389/fmedt.2022.884314 (PMC9108230; doi:10.3389/fmedt.2022.884314)
Supplement: Supplementary file 1 [file Data_Sheet_1.docx]

Supplementary Material

# Supplemental Methods

## Finite Element Modeling of Hydrogel Composite

To investigate the impact of compression on spatial differences in hydrogel stress of GelMe hydrogels, a finite element model of compression was developed using the FEBio software suite (https://febio.org). A cylindrical model was created mimicking the form of the hydrogel with 8 mm diameter and 2 mm height. The density of the GelMe was chosen to be 1.02 g/cm^3^. The Young’s Modulus of the model directly mimicked the 7 kPa modulus of the hydrogel with a poison ratio of 0.49. The bottom of the model was chosen to be fixed in x,y, and z since the GelMe hydrogel was attached to the bottom of a well in a 24-well plate. Two pressures were used representing the force applied by the compressive device on the model. Resulting from the trial-and-error method, 1,230 Pa was chosen to represent 25% of the compressed height of the model and 2,370 Pa represented 50% of the compressed height of the model. Figure 2 shows the model (Fig. 2A). The simulation was done in the highest time step possible to represent the ultimate stress applied.

# Supplementary Figures and Tables


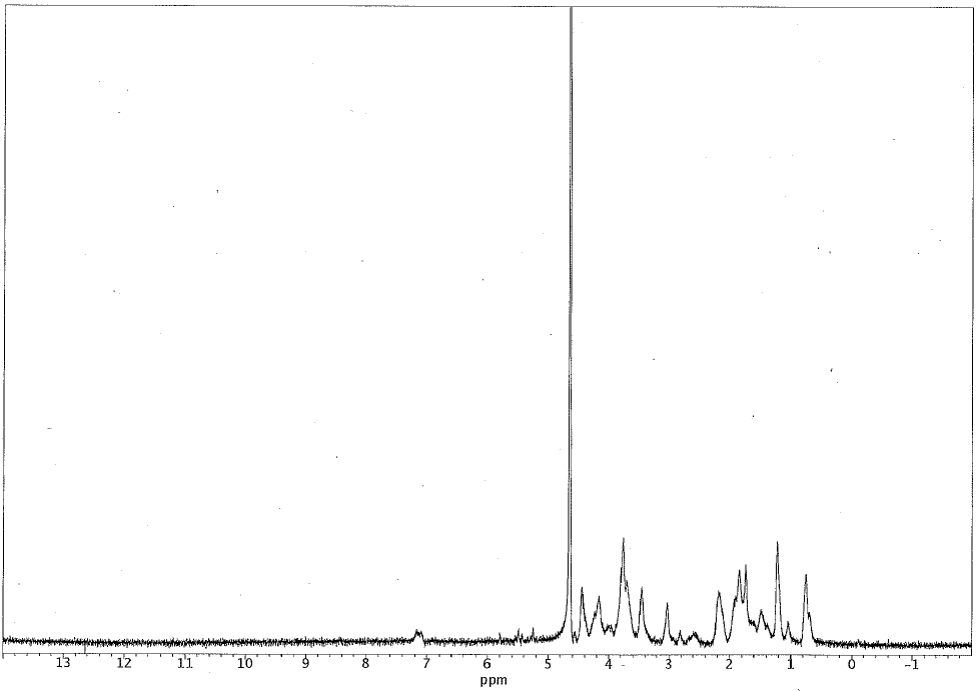


**Supplementary Figure 1.** ^1^H NMR spectroscopy of methacrylated gelatin shows a 58.2% modification with methacrylates.


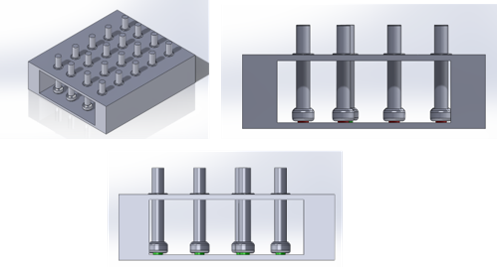


**Supplementary Figure 2.** SolidWorks model of the custom compression device.

**
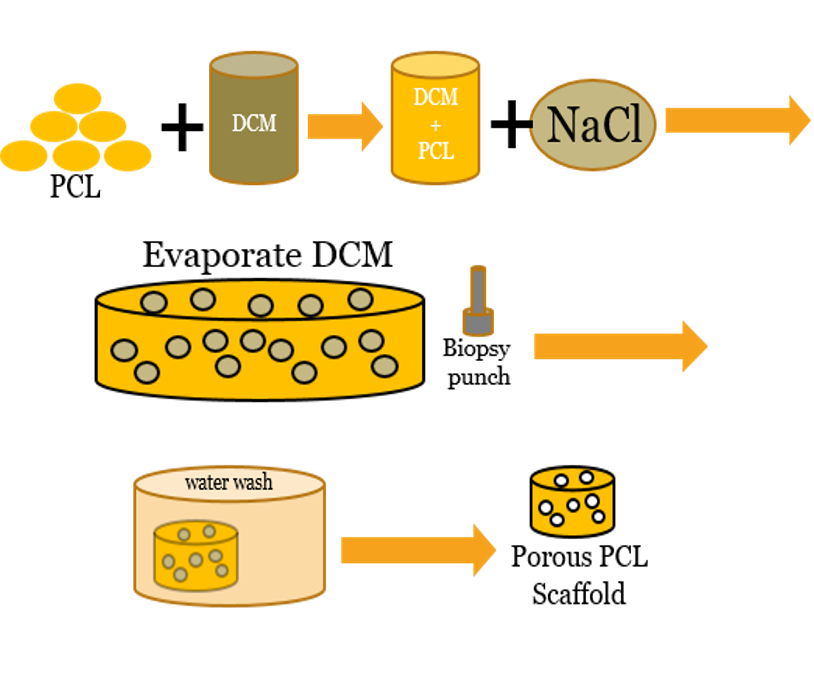
**

**Supplementary Figure 3.** Schematic showing the formation of porous PCL scaffolds. First, PCL is dissolved in DCM then the PCL solution is mixed with salt. Next, the DCM is evaporated overnight and an 8 mm biopsy punch is used to create cylindrical structures. Lastly, the constructs are washed with water to leach the salt resulting in porous PCL scaffolds.


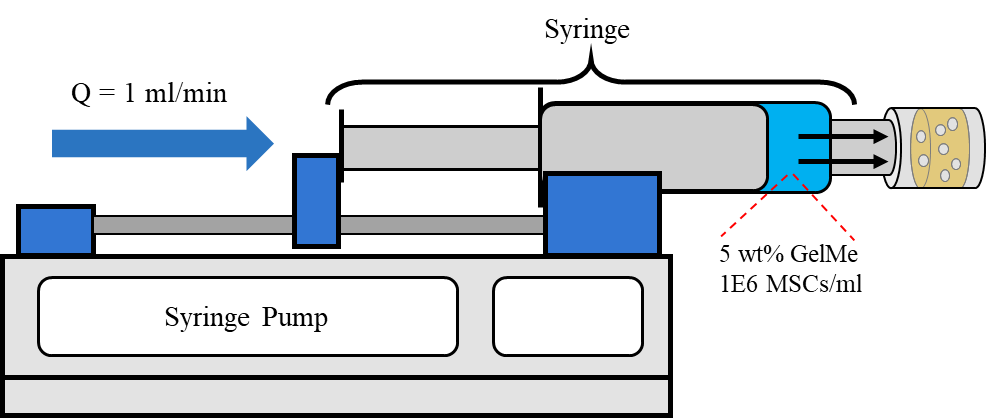


**Supplementary Figure 4.** Schematic for perfusing MSC-laden GelMe solution into porous PCL scaffolds. A syringe containing hydrogel solution (5 wt% GelMe, 1E6 MSCs/ml, 0.05 wt% I2959) is placed on a syringe pump. The tip of the syringe is connected to a tube containing a porous scaffold. To perfuse hydrogel solution into the porous scaffold, the syringe pump extrudes the hydrogel solution at a rate of 1 ml/min.


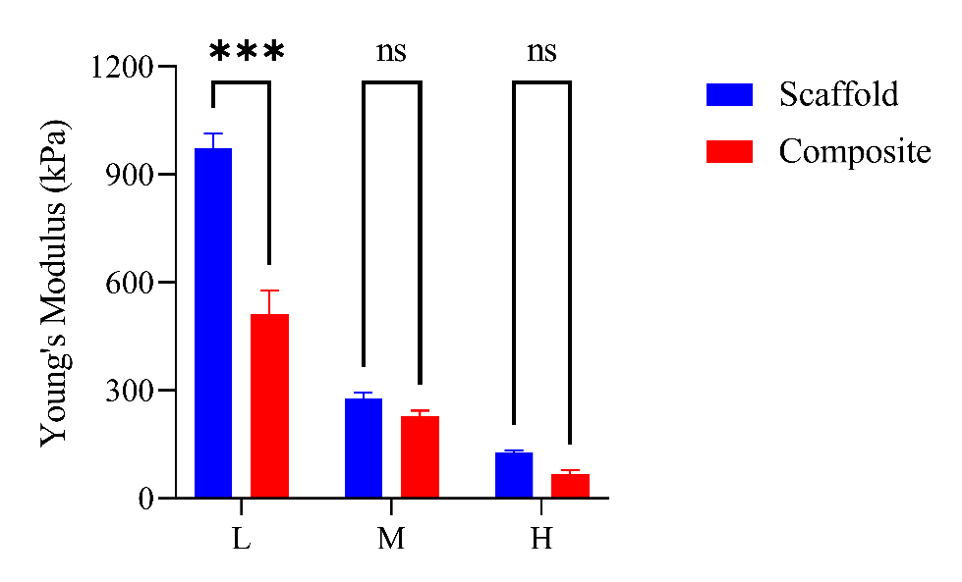


**Supplementary Figure 5.** The Young’s Modulus of PCL scaffolds and PCL scaffold-GelMe hydrogel composites. The hydrogel alone has a Young’s Modulus of 7.2 ± 0.2 kPa. Bar graphs represent the mean and error bars standard error of the mean, ns not significant, *** p < 0.001.


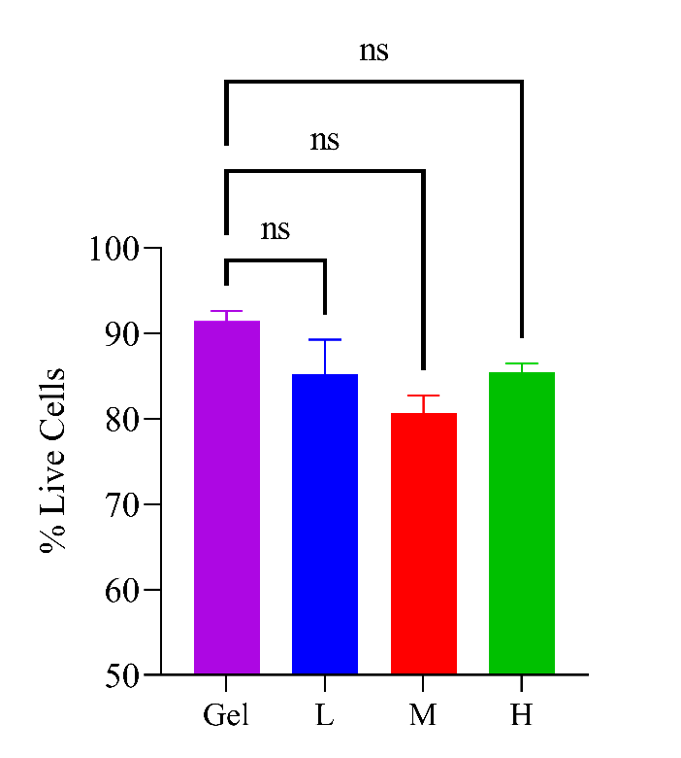


**Supplementary Figure 6.** Cell viability of encapsulated MSCs in GelMe hydrogel and composites with L, M, and H pore sizes. MSC viability is ≥ 80% across all groups. Bar graphs represent the mean and error bars standard error of the mean, ns not significant.
